# Rethinking how research is reviewed and published

**DOI:** 10.7554/eLife.110392

**Published:** 2026-03-17

**Authors:** Timothy E Behrens, Yamini Dalal, Diane M Harper

**Affiliations:** 1 eLife Cambridge United Kingdom

**Keywords:** scientific publishing, peer review, preprints, research assessment, publish-review-curate

## Abstract

Taking a radical new approach to the publication process resulted in eLife losing its impact factor, but authors, reviewers, editors and funders support the journal and its efforts to reform scientific publishing.

In 2023, eLife moved to a new model of publishing in which all articles that are peer reviewed are published on the eLife website, along with feedback from the reviewers. Moreover, every article now includes an eLife Assessment, written by the editor and reviewers, that summarises the significance of the findings reported in the article and the strength of the evidence. Since eLife only reviews articles that are available on a preprint server, this new approach to publishing combines the speed and openness of preprints with the scrutiny of expert peer review.

The aim of this approach – which is an example of the publish-review-curate (PRC) model – is twofold. First, to move to a system in which research articles are judged on the basis of their scientific content, not on the name or impact factor of the journal in which they are published. This means that all readers – including panels and committees charged with evaluating scientists for jobs, tenure and promotion – can engage easily and meaningfully with the strengths and weaknesses of the research being reported.

Second, to move to a system where authors do not have to cascade down a hierarchy of journals until they find favourable reviewers. Instead, if an article is peer reviewed by eLife, the authors know that it will be published, and they do not have to worry about going through the whole process again at a different journal. Authors can also decide when to publish their work as a regular journal article (the Version of Record) to mark the end of the review and publication process. That said, in our experience, almost all authors revise their articles in response to the comments from the reviewers.

In the three years since we changed eLife’s approach to publishing, we have learnt a great deal. We should, for example, have explained sooner how and why we select papers for peer review. We also underestimated the commercial forces that the scientific community as a whole will need to overcome to enact any meaningful change in publishing. Overall, however, the most important thing we have learnt is that our new approach to publishing works. Authors, reviewers and editors routinely tell us that they have had a more constructive experience with the new approach.

Earlier this month we announced that Wellcome has invested £2.4m over three years in eLife Pathways, our initiative to build open publishing infrastructure for the global research community. Today, we are releasing submission and review data for the period from February 2023 until the end of 2025. The story told by the data is complex, but in our view remarkable. Two major changes took place during this period: we introduced our version of the PRC approach and, at the end of 2024, Clarivate – the company responsible for the impact factor – announced that our new approach would mean that we would lose our impact factor.

To our knowledge, it is unprecedented for a major international journal to lose its impact factor for reasons other than editorial misbehaviour, such as mass self-citation. Such journals tend to suffer submission and editorial board collapse. What would happen if a journal lost its impact factor for its values, rather than its behaviour? Would we too collapse? If so, can any journal ever innovate without the permission of the powerful commercial sector?

To cut a long story short, the loss of our impact factor did lead to a drop in submissions, but this fall was smaller than the falls seen at other journals that have lost their impact factor. There are countries that still rely heavily on the impact factor for scientific evaluation, and submissions from these countries were particularly affected. Moreover, like all journals, eLife has always received a number of low quality submissions. We now receive fewer of these, which means that we are currently reviewing a higher proportion of submissions (around 35% in 2025, compared with 27% in 2024). Most exciting, we have a substantial author base who remain with us, who are choosing eLife not because of our impact factor, but because of our values and our review process. Overall, we reviewed and published an average of 84 articles per month in 2025, compared with 143 in 2024.

Critically, this number has been fairly stable for a year. Moreover, we can measure the quality of the articles we publish in a quantitative manner because, during the review process, we ask reviewers to choose terms that assess the significance of the findings and the strength of evidence in the article. We also did this for the last year of our old model. We have compared the distributions of the terms chosen by reviewers for that year, the period before the Clarivate decision, and the period after, and found them to be remarkably similar.

Having proven that the PRC approach works, we will now focus our energy on making it the best system possible for communicating science and the discussion around science. This means that we will stop accepting new submissions to our legacy peer-review process, but we will continue to bring new developments to PRC.

Although every eLife article contains an eLife Assessment that summarizes what the reviewers thought about the article, there is a lot more we could do to enhance the discussion around articles and help readers engage with our reviews. Modern AI tools offer an opportunity to do this, and we are partnering with a company called qed science to develop new approaches. We are also going to reintroduce the option for reviewers to be named so that they can receive public recognition for their work.

Similarly, we want to explore new ways for authors to communicate their scientific message. We are particularly excited by a recent addition whereby authors can embed explainer videos in any, or every, figure in an article, giving the reader the experience of being at a seminar, whilst also being able to read the details of the article. (Please see our author guide for more details, and this article for examples of explainer videos.) In this new world there will also be opportunities to broaden the types of studies we can publish. For example, we recently introduced a new article type specifically for Replication Studies. One reason other journals may be reluctant to promote such articles is because they want to protect their impact factor!

Scientific publishing must evolve for the modern era. It must promote science but also provide a framework for honest discourse around science. The last three years at eLife suggest such a future is possible, while maintaining high standards of peer review and editorial judgment. It shows that even when commercial forces need to be confronted, a substantial community of authors subscribe to this future, and in doing so they benefit from a more constructive experience.

